# Vestigialization of an Allosteric Switch: Genetic and Structural Mechanisms for the Evolution of Constitutive Activity in a Steroid Hormone Receptor

**DOI:** 10.1371/journal.pgen.1004058

**Published:** 2014-01-09

**Authors:** Jamie T. Bridgham, June Keay, Eric A. Ortlund, Joseph W. Thornton

**Affiliations:** 1 Institute of Ecology and Evolution, University of Oregon, Eugene, Oregon, United States of America; 2 Biochemistry Department, Emory University School of Medicine, Atlanta, Georgia, United States of America; 3 Departments of Human Genetics and Ecology & Evolution, The University of Chicago, Chicago, Illinois, United States of America; University of Michigan, United States of America

## Abstract

An important goal in molecular evolution is to understand the genetic and physical mechanisms by which protein functions evolve and, in turn, to characterize how a protein's physical architecture influences its evolution. Here we dissect the mechanisms for an evolutionary shift in function in the mollusk ortholog of the steroid hormone receptors (SRs), a family of biologically essential transcription factors. In vertebrates, the activity of SRs allosterically depends on binding a hormonal ligand; in mollusks, however, the SR ortholog (called ER, because of high sequence similarity to vertebrate estrogen receptors) activates transcription in the absence of ligand and does not respond to steroid hormones. To understand how this shift in regulation evolved, we combined evolutionary, structural, and functional analyses. We first determined the X-ray crystal structure of the ER of the Pacific oyster *Crassostrea gigas* (CgER), and found that its ligand pocket is filled with bulky residues that prevent ligand occupancy. To understand the genetic basis for the evolution of mollusk ERs' unique functions, we resurrected an ancient SR progenitor and characterized the effect of historical amino acid replacements on its functions. We found that reintroducing just two ancient replacements from the lineage leading to mollusk ERs recapitulates the evolution of full constitutive activity and the loss of ligand activation. These substitutions stabilize interactions among key helices, causing the allosteric switch to become “stuck” in the active conformation and making activation independent of ligand binding. Subsequent changes filled the ligand pocket without further affecting activity; by degrading the allosteric switch, these substitutions vestigialized elements of the protein's architecture required for ligand regulation and made reversal to the ancestral function more complex. These findings show how the physical architecture of allostery enabled a few large-effect mutations to trigger a profound evolutionary change in the protein's function and shaped the genetics of evolutionary reversibility.

## Author Summary

An important goal in evolutionary genetics is to understand how genetic mutations cause the evolution of new protein functions and how a protein's structure shapes its evolution. Here we address these questions by studying a dramatic lineage-specific shift in function in steroid hormone receptors (SRs), a physiologically important family of transcription factors. In vertebrates, SRs bind hormones and then undergo a structural change that allows them to activate gene expression. In mollusks, SRs do not bind hormone and are always active. We identified the genetic and structural mechanisms for the evolution of constitutive activity in the mollusk SRs by using X-ray crystallography, ancestral sequence reconstruction, and experimental studies of the effects of ancient mutations on protein structure and function. We found that constitutive activity evolved due to just two historical substitutions that subtly stabilized elements of the active conformation, and subsequent mutations filled the hormone-binding cavity. The structural characteristics required for a hormone-sensitive activator were thus vestigialized, much the same way that a whale's hindlimbs became vestiges of their ancestral form after they became dispensable. Our findings show how the architecture of a protein can shape its evolution, allowing radically different functions to evolve by a few large-effect mutations.

## Introduction

Understanding the mechanisms by which protein functions evolve is a major goal in studies of molecular evolution. A question of particular interest is how the biophysical architecture of a protein shapes its evolutionary potential. This question is a specific form of a general issue long-discussed by evolutionary biologists: whether and how the structure of complex biological systems – the process of organismal development or physiology, for example – influences evolution's capacity to modify those systems and produce new phenotypes [1]–[3]. This issue is typically discussed in terms of constraints on evolution, which are conceived as limits placed on the types of phenotypic variation that can be produced through mutation. In principle, however, the structure of biological systems could also act positively, facilitating the evolution of certain new phenotypes via simple genetic changes [4].

Proteins, although small in scale, are also complex biological systems, because their functional behavior is determined by specific interactions between large numbers of differentiated elements – in this case, thousands of atoms in three-dimensional space. How a protein's structure determines its evolutionary potential has been studied to only a limited extent. Prior work on suggests general patterns of constraint imposed by structure and function: residues in the protein core are generally less amenable to substitution than those on the surface [5]–[7], and interacting residues in proteins can exert site-specific constraints on each other [7]–[9]. To understand how structure affects the evolution of new functions, however, it is necessary to directly trace the mechanisms by which functional changes occurred during historical evolution.

Vertical approaches that experimentally dissect historical evolution through time are particularly useful, because they elucidate the genetic and structural mechanisms by which historical shifts in protein function took place. In cases of very recent functional evolution, population genetic methods can be used to identify which sequences, functions, and structures are ancestral and which are derived [10]–[12]. For more ancient divergences, ancestral protein reconstruction (APR) allows the functions and structures of ancient proteins to be experimentally characterized and provides a platform for identifying the historical mutations that mediated shifts in these properties [13], [14]. In only a few cases have historical shifts in function been analyzed using experimental genetic and structural analysis of ancestral proteins [4], [15]–[18], although several additional studies have attempted to model ancestral structures based on their extant descendants [19]–[22]. In virtually all of these cases, the shift in function under study was a relatively subtle change in specificity. How fundamental changes in function evolve, such as the gain/loss of a biochemical activity or mode of regulation, remains largely unstudied (but see ref. [19]).

An ideal group of proteins for studying the structural mechanisms of functional evolution would be functionally diverse, contain adequate phylogenetic signal for reconstructing ancestral protein sequences and their historical mutational trajectories, and have well-developed methods for functional and structural characterization. The steroid receptor (SR) protein family fulfills these criteria in general [16]–[18]; in this paper, we focus on the mechanisms for a lineage-specific change in the mode of allosteric regulation by ligands in the SR ortholog of mollusks.

In vertebrates [23] and some invertebrates [24], [25], members of the SR gene family are hormone-activated transcription factors, which regulate developmental, reproductive, and physiological processes. The protein's ligand-binding domain (LBD) serves as an allosteric switch controlled by the hormone, which binds in an internal hydrophobic cavity deep in the protein's LBD; ligand binding shifts the domain's thermodynamic equilibrium from the inactive conformation when ligand is absent to the active conformation when ligand is bound. In the inactive conformation, a C-terminal “activation-function” helix (AF-H) is disordered or extended away from the rest of the protein. In the active conformation, AF-H packs against the body of the protein, contributing to the assembly of a new surface that attracts coactivator proteins that alter chromatin or otherwise potentiate transcription of nearby target genes. Ligand binding stabilizes the position of the other helices against which AF-H packs and thus increases the stability of the active conformation relative to the inactive conformation [26], [27].

In contrast to vertebrate SRs, mollusks contain a single SR ortholog that is unique in being a ligand-independent constitutive transcriptional activator. (Because the mollusk receptors are most similar in sequence to the estrogen receptors of vertebrates, they are commonly referred to as ERs, although the phylogenetic analyses reported in this paper indicate that they are equally orthologous to the entire clade of vertebrate SRs.) The ligand-binding domains from diverse mollusk ERs, including those of the sea slug *Aplysia californica*, the cephalopod *Octopus vulgaris*, the clam *Thais clavigera*, and the oyster *Crassostrea gigas*, have all been shown experimentally to activate transcription at high levels in the absence of any added ligands; further, they do not bind estrogens, and no increase in transcriptional activation is observed when the receptor is treated with hormones or other substances [28]–[31]. This constitutive function is thought to be evolutionarily derived, because the LBDs of other invertebrate SRs – including two from species in the closely related phylum of annelid worms, as well as the cephalochordate *Branchiostoma floridae* – lack constitutive activity and can be activated by addition of estrogens [24], [25]. Further, phylogenetic reconstruction, synthesis, and experimental characterization of the ancestral gene from which the entire SR family descends (AncSR1) showed that AncSR1 was estrogen activated with very little constitutive activity [28], [32].

The mechanisms by which the mollusk ERs' constitutive transcriptional activity evolved are unknown. No structures of these proteins are available, and there has been no genetic or evolutionary work identifying the key substitutions that confer on these proteins their unique functions. Numerous questions are therefore unanswered: whether this derived function required many or few mutations, whether it was brought about by additive contributions from historical sequence changes or by a complex of epistatically interacting mutations, and what sort of remodeling of the protein structure was required to confer it. Constitutive activity has been observed in some distantly related members of the nuclear receptor superfamily; comparing crystal structures among constitutive and non-constitutive receptors suggests that the underlying mechanisms are diverse. In some cases, the transcriptionally active conformation appears to be stabilized in the absence of ligand by the acquisition of bulky residues that fill the hydrophobic cavity [33], [34]. In others, electrostatic interactions between side chains or improved packing interactions between structural elements in the protein appear to stabilize the active conformation [35], [36]. In still others, an omnipresent ligand fills the pocket, causing activity even when no exogenous ligand is added and suggesting ligand-independence before this structural information was available [37]. No studies, however, have identified the historical mutations that caused constitutive activity to evolve.

Here we characterize the evolution of the mollusk ER's lineage-specific function by combining structural and genetic analysis of the constitutive ER of the oyster *Crassostrea gigas* with experimental reconstruction, manipulation, and characterization of ancestral proteins in the lineage leading to mollusk ERs. This combination of structural and evolutionary genetic approaches allows us to analyze in detail the mechanisms by which receptor function evolved and how the protein's structure shaped its functional and genetic evolution.

## Results and Discussion

### The ligand pocket of mollusk ER is occluded

**Figure 1:**
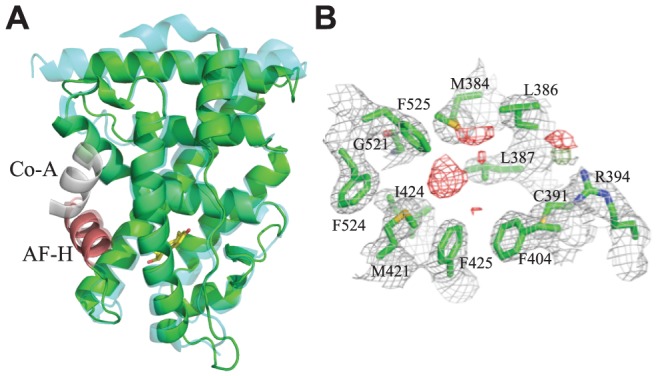
Crystal structure of CgER in the active conformation without ligand. **A.** The X-ray crystal structure of CgER LBD (green) was determined in the absence of added ligand. CgER is shown superposed on the backbone of the X-ray crystal structure (PDB 1GWR) of human ERα (cyan) in complex with estradiol (yellow sticks) and coactivator peptide (co-A, white). The activation function helix (AF-H) is red. **B.** Lack of electron density for ligand in the crystallized CgER. Side chains of residues that form the ligand-binding pocket are shown as green sticks, amino acids are numbered by position in the human ERα sequence. 2F_O_ – F_C_ electron density is shown in grey, contoured at 1σ. F_O_ – F_C_ electron density is shown at σ = ±2.5, with positive values shown in green and negative values shown in red.

To understand the mechanisms by which constitutive activity evolved in the *Crassostrea gigas* ER (CgER), we used X-ray crystallography to determine the three-dimensional structure of its LBD at a resolution of 2.6 Å in the absence of any added ligands (Table S1, Fig. 1A). The domain is in the classic active conformation, and the backbone conformation is similar to that of the human ERα (1.36 Å RMSD for all atoms).

**Figure 2:**
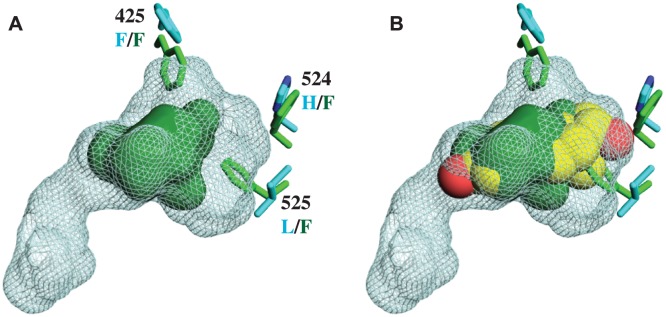
CgER ligand binding pocket is too small to accommodate estradiol. **A.** The ligand cavity in the human ERα (1GWR, cyan mesh) is much larger than the corresponding volume in CgER (solid green). Side chains of residues that occlude the cavity in CgER are shown as sticks; residue numbers are from human ERα, with states in CgER and human ERα labeled in green and cyan text, respectively. **B.** The CgER cavity is too small to accommodate estradiol (yellow spheres, positioned as in 1GWR).

Unlike other SRs previously studied, there is no electron density of a ligand in the interior of the CgER LBD (Fig. 1B). Rather, the internal cavity where ligand binds in other steroid receptors is occupied in CgER by several bulky hydrophobic side-chains, including F425 and F525, and – to a lesser extent – F524 (using human ERα numbering to facilitate comparison). These hydrophobic residues would strongly clash with estradiol as it is oriented in the human ERα LBD (Fig. 2). The resulting cavity has a total volume (168±8 Å^3^) much smaller than that of human ERα (402 Å^3^) and too small to accommodate estradiol (245 Å^3^) and other steroids.

This structure has several implications. First, it indicates that CgER is an authentically ligand-independent transcriptional activator, which exists in the active conformation in the absence of ligand or other apparent modifications. If estrogen-like compounds, endogenous or environmental, affect mollusk reproduction and physiology, as has been reported previously [38]–[42], our findings indicate that these impacts must be mediated by mechanisms other than ER activation.

Second, the presence in the protein interior of bulky residues that occlude the ligand cavity provides a physical rationale for the inability of mollusk ERs to bind or be activated by ligands [28]–[31]. Although we cannot rule out the possibility that some unknown substances might be bound by the mollusk ER, the extremely small size of the cavity when the protein is in the active conformation suggests that if any such ligands exist, they would have antagonist rather than agonist effects on transcriptional activation.

Third, the structure confirms that the constitutive activity of mollusk ERs is a derived evolutionary character, because no other members of the steroid receptor family have filled ligand cavities. A more distantly related clade within the nuclear receptor superfamily – the ERRs – are constitutive activators with partially filled internal cavities, but the residues that fill the pocket are at different sites in the sequence [34], [43].

### Reconstruction of the ancestral lophotrochozoan SR

The CgER structure alone is insufficient to determine the mechanisms by which constitutive activity evolved. Ligand-independent activity may have been caused by the mutations that filled the receptor's ligand pocket; alternatively, it may have been caused by genetic changes that stabilized the active conformation by different mechanisms, followed by the substitutions that occluded the cavity.

**Figure 3:**
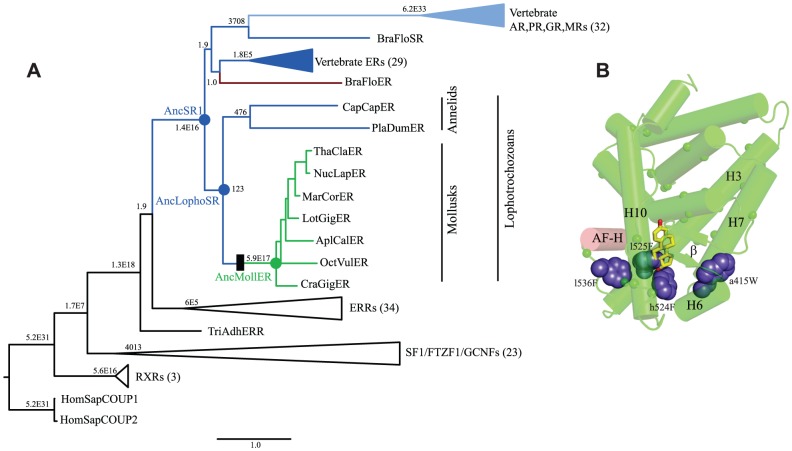
Constitutive activity evolved on the branch between ancestral lophotrochozoan and mollusk steroid receptors. **A.** Phylogeny of steroid receptors and related proteins. The maximum likelihood phylogeny of 135 protein sequences is shown; clades containing multiple closely related proteins are shown as wedges with the number of sequences in parentheses. Branch labels show statistical support for each node as the approximate likelihood ratio in scientific notation. Members of the SR clade are colored by mode of allosteric regulation: receptors activated by estrogen or other steroids are colored dark or light blue, respectively; constitutively active transcription factors are green; proteins that act as dominant negative regulators of transcription are red. Scale bar, substitutions per site. Black box represents evolutionary gain of constitutive transcriptional activation and loss of ligand binding. Ancestral proteins referred to in the text are labeled as AncSR1 (ancestral steroid receptor), AncLophoSR (SR in the ancestor of lophotrochozoan species), and AncMollER (ER in the ancestor of mollusks). Exant species abbreviations are: AplCal, *Aplysia californica*, BraFlo, *Branchiostoma floridae*, CapCap, *Capitella capitata*, CraGig, *Crassostreas gigas*, HomSap, *Homo sapiens*, LotGig, *Lottia gigantia*, MarCor, *Marisa cornuarietis*, NucLap, *Nucella lapillas*, OctVul, *Octopus vulgaris*, PlaDum, *Platynereis dumerilii*, ThaCla, *Thais clavigera*, TriAdh, *Trichoplax adhaerens*. **B.** Candidate causal substitutions for the shift in allosteric regulation in the mollusk steroid receptors. Residues that changed between AncLophoSR and AncMollER and are conserved in all or all but one extant mollusk ER are indicated by small spheres for Cα carbons. Of these, four (shown as large blue spheres) occupy positions close to the ligand cavity of other SRs. AF-H is red; estradiol, as positioned in the human ERα structure (1GWR), is shown as yellow sticks.

To identify specific historical substitutions that caused constitutive activity to evolve in the mollusk ERs, we identified candidate mutations that occurred during the historical interval in which constitutive activity emerged and tested them in the context of the relevant ancestral sequence. We began by reconstructing the amino acid sequence of the SR protein as it existed in the last common ancestor of the various lophotrochozoan phyla, including annelids and mollusks. We aligned 135 present day receptor sequences from a wide range of invertebrates and vertebrates (Table S2), determined the best-fit evolutionary model, and inferred the maximum likelihood phylogeny (Fig. 3A, Fig. S1). This phylogeny is largely consistent with the results of previous analyses [18], [28], [29], except that in this case the lophotrochozoan ERs are not a sister group to the chordate ERs but are instead placed outside of all chordate SRs, including the vertebrate ERs, androgen receptors (ARs), progestagen receptors (PRs), glucocorticoid receptors (GRs), and mineralocorticoid receptors (MRs). This topology is more parsimonious than the previous one, because it implies a single receptor in the ancestor of all bilaterally symmetric organisms, followed by the minimum possible number of gene duplications – all in the chordates – and no subsequent losses. In contrast, placing the lophotrochozoan ERs as sister to the chordate ERs requires an additional earlier gene duplication and a subsequent loss of the AR/PR/GR/MR group from the lophotrochozoans. The present analysis is also based on more complete taxonomic sampling than previous efforts and therefore represents the best current hypothesis of SR phylogeny.

To test the hypothesis that ligand-independent activation evolved on the branch leading to the ancestral mollusk ER, we inferred the maximum likelihood sequence of the ancestral lophotrochozoan SR (AncLophoSR); this is the reconstructed sequence at the node in the phylogeny that represents the SR ortholog in the last common ancestor of all extant lophotrochozoans (Fig. 3). AncLophoSR is 58.4% identical to CgER-LBD, and 57.9% identical to the Human ERα (Fig. S2). Support for the reconstruction was only moderate (Fig. S3): the mean posterior probability over all sites was only 0.73, with 35 sites with a plausible alternate reconstruction (defined as a second-best state with PP>0.25) (Table S3). At the 16 sites that line the internal cavity where ligands bind, however, confidence is a higher, with a mean PP of 0.91 and only one ambiguously reconstructed site.

We tested the ligand-dependence of the AncLophoSR LBD by expressing it as a Gal4-DBD fusion protein and characterizing its transcriptional activity using a reporter gene assay in transfected cultured cells. In contrast to the constitutively active mollusk ERs, AncLophoSR had virtually no ligand-independent transcriptional activity, and it exhibited a clear dose-responsive increase in activity as estrogen concentrations were increased. AncLophoSR is highly sensitive to estradiol with an EC50 of 12 nM, although its maximal activation in this assay is lower than that of the human ERα [29] (Fig. S4).

We next sought to determine the sensitivity of this result to uncertainty about the ancestral sequence. The number of sites with alternative plausible reconstructions is too large for us to test them all individually. We therefore synthesized a radically different version of AncLophoSR containing all 35 plausible alternative amino acids. Although this sequence is far less likely to be correct than the ML reconstruction (with a likelihood that is 2 million times lower), it represents the “far edge” of the cloud of plausible ancestral reconstructions and allows a very conservative test of the robustness of inferences about the ancestral protein's functions. When expressed and assayed, this sequence was also estrogen-activated, although its baseline activity was somewhat higher than that of AncLophoSR-ML (Fig. S5). This result indicates that the estrogen-sensitivity of AncLophoSR is robust to statistical uncertainty about the ancestral reconstruction. Taken together, these findings corroborate the conclusion, supported by other lines of evidence, that ligand-independence evolved in the mollusks from an estrogen-sensitive ancestral state [18], [24], [25], [28].

### Identifying historical substitutions

To identify the historical sequence changes that caused the evolution of constitutive activity and the loss of ligand regulation, we combined phylogenetic and structural analysis. These events must have occurred on the branch of the phylogeny leading from AncLophoSR to the ancestral mollusk ER, because all mollusk ERs are constitutively active and ligand-insensitive [28]–[31]. Seventy-nine amino acid replacements took place on this branch.

To narrow down the list of candidate substitutions for a causal historical role, we reasoned that functionally important residues are most likely to be conserved among descendant sequences and to be located in the regions of the protein structure that differ between the mollusk ERs and other SRs. Of the 79 substitutions, 44 are conserved in all or all but one mollusk ER sequences (Fig. S6). We plotted these historical replacements on the crystal structures of CgER and human ERα and on a homology model of AncLophoSR. Four historical sequence changes emerged as top causal candidates, because they contributed to filling the ligand pocket or improved packing among helices near the pocket and/or the coactivator interface.

These four sites form a ring around the lower portion of the ligand cavity in the elements of the structure that are stabilized by interactions with the ligand in vertebrate SRs. Specifically, a415W (on the loop between helices H6 and H7), h524F and l525F (both on H10), and l536F (on the loop between H10 and AF-H) place large hydrophobic side chains into open spaces within the ligand cavity or in smaller spaces between key helices (Fig. 3B, using lower and upper case to denote the states in AncLophoSR and CgER, respectively).

### Genetic causes of the evolution of constitutive activity

**Figure 4:**
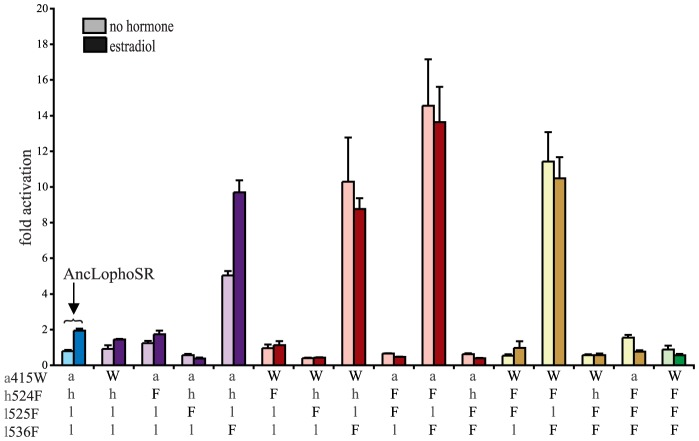
Two historical substitutions recapitulate the evolution of constitutive activity. Hormone-dependent transcriptional activation by the ancestral lophotrochozoan SR (AncLophoSR) and mutants containing all possible combinations of ancestral or derived states at four candidate sites was assessed using a dual luciferase assay. Lower case letters denote ancestral states; derived states in the mollusk ER are shown as upper case. Colors indicate number of residues with derived states: blue (zero), purple (one), red (two), yellow (three), and green (four). Fold activation (luciferase activity relative to vector-only control) is shown as mean and SEM over three experiments with three replicates each.

To test the hypothesis that these four sequences changes conferred on the evolving mollusk ER its constitutive activity, we introduced the CgER states into the AncLophoSR background and determined their impacts on activation and allosteric regulation by ligand. When introduced singly, none of the four was sufficient to fully recapitulate the derived phenotype. l536F, however, caused a very large increase in constitutive activity (and on maximal ligand-dependent activity, as well). Neither a415W and nor h524F had an observable effect on its own. l525F abolished all activity, indicating that it is incompatible with the ancestral background (Fig. 4).

We next assayed all possible two-fold combinations of the four candidates. We found that two pairs – h524F/l536F and a415W/l536F – each completely recapitulated evolution of the CgER-like phenotype, with very high constitutive activity and no additional activity induced by ligand. Despite the strong contributions of h524F and a415W when combined with l536F, the pair a415W/h524F had little effect on either constitutive or ligand-induced activity. All combinations containing l525F abolished both types of activity.

These data indicate that large-effect mutations played key roles in the evolution of constitutive activity, with just two substitutions required to recapitulate the entire shift in function in the likely ancestral background. Further, significant epistasis is present, because the effects of h524F or a415W differ radically depending on whether site 536 has the ancestral leu (in which case these substitutions have no apparent effect) or the derived Phe (in which case they abolish ligand-dependent activity and yield a solely constitutive activator) (Fig. 4).

We next prepared all three-fold combinations and found that combining the three substitutions that in pairs contribute to the derived phenotype – h524F, a415W and l536F – causes no functional difference compared to h524F/l536F or a415W/l536F (Fig. 4). Epistasis is again apparent in the redundant effects of these mutations on function: adding either h524F or a415W to l536F abolishes ligand-regulation and further enhances constitutive activity, but adding both causes the same effect as adding either one. We therefore conclude that two historical amino acid replacements from the mollusk lineage are sufficient to recapitulate the evolution of constitutive activity in the ancestral background: l536F makes a major, independent contribution, and adding either h524F or a415W is sufficient to explain the evolution of total ligand-independent activity.

The fourth candidate substitution – l525F – abolishes all transcriptional activity, both constitutive and ligand-dependent, when introduced in isolation or in any combination with the other substitutions (Fig. 4). This result is surprising, because the derived state F525 is conserved among all known mollusk ERs and does not render them nonfunctional. Other historical mutations that occurred in the stem mollusk lineage must interact epistatically with l525F, exerting a permissive effect that allows mollusk ERs to tolerate l525F without losing function.

The residues found in CgER at three of these four sites – W415, F525, and F536 – are conserved among all known mollusk ERs. The fourth, F524 in the CgER, is a tyrosine in the ancestral mollusk ER and most extant mollusks, suggesting that mutation Y524F occurred later in the lineage leading to *Crassostrea*. To determine the effect of having a tyrosine at this position, we repeated all experiments using genotypes containing tyrosine instead of phenylalanine at site 524. In every background, the tyrosine yielded nearly identical functional behavior as the phenylalanine (Fig. S7), indicating that either of the two bulky aromatic states found in mollusk ERs can make a similar contribution to the evolution of constitutive activity. Taken together, these data indicate that two historical substitutions – l536F and either a415W or h524Y – were sufficient to cause the evolution of constitutive activity, and the subsequent substitution Y524F in the lineage leading to CgER did not affect this activity.

### Reversing the evolution of constitutive activity in CgER

**Figure 5:**
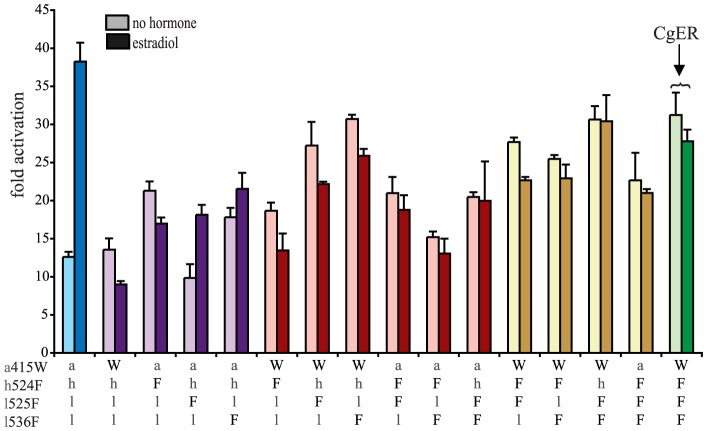
Reversing four historical substitutions in CgER restores estrogen sensitivity. All combinations of derived and ancestral states at the four candidate sites were introduced into CgER and their effects on activation in the presence or absence of hormone was characterized in a luciferase reporter assay. Colors indicate number of residues reversed to ancestral states: green shows the CgER protein (zero ancestral states), yellow (one ancestral state), red (two), purple (three), blue (all four). Lower case letters denote ancestral states; mollusk derived states are shown as upper case.

Given the fact that two pairs of three historical substitutions are sufficient to recapitulate the evolution of constitutive activity in AncLophoSR, we asked whether reversing them to their ancestral states in CgER could restore the ancestral ligand-regulated function. The answer is no. When either pair is reversed, full constitutive activity remains present and no ligand regulation is apparent. This result indicates that additional “restrictive” mutations occurred, which made reversal of the mutations that were once sufficient to cause the evolution of the new function no longer sufficient to restore it [16]. The three-fold revertant also remained fully constitutive and ligand-independent. (Fig. 5)

When l525F, the fourth candidate substitution near the ligand pocket, was also reversed, however, the four-fold revertant became ligand-regulated, manifesting a >3-fold, dose-responsive increase in activation upon administration of estradiol (Figs. 5, S4B). This change is accomplished by knocking down constitutive activity substantially while maintaining high levels of activation only when hormone is added. Although more than 90 other replacements occurred between AncLophoSR and CgER, reversing just four of them is therefore sufficient to restore an allosteric response to hormone. Additional derived states in CgER must have had a further restrictive effect, because reversing these four substitutions does not fully abolish constitutive activity.

We next determined whether it is necessary to reverse all four states to restore ligand dependence to CgER by assaying all possible combinations of the four ancestral and derived residues in the CgER protein. The answer is yes: only when all four states are reversed is full ligand-dependent activity restored, and just one of the triple revertants displayed even partial ligand-dependent activation (Fig. 5). Thus, although only two of the four historical substitutions are necessary to trigger the evolution of ligand-independent activity, two others must also be reversed for the ancestral function to be reacquired. Specifically, the historical pairs l536F/a415W or l536F/h524F are sufficient to yield constitutive activity; whichever pair came first, adding the third redundant substitution has no further effect on function but prevents reversing the other two from restoring the ancestral function. The fourth substitution, l525F, is also restrictive, but instead of having no effect on function, it requires permissive mutations to be tolerated. These mutations' effects are unlike those of previously observed restrictive mutations that impede irreversibility [16]: in those cases, the restrictive substitutions created a genetic background in which reverting the function-switching mutations to the ancestral state renders the protein nonfunctional. In the mollusk ER, the restrictive substitutions cause reversal of the key substitutions to be functionally inconsequential, not deleterious.

These inferences about reversibility are robust to uncertainty about the ancestral states. At three of the four key sites, the ancestral state in AncLophoSR are reconstructed with little or no ambiguity. At the other – ala415 – serine is a possible alternate state, with PP = 0.20. Reversing Trp415 in CgER to ser instead of ala along with the three other ancestral states also restores ligand-sensitive activity to CgER. Moreover, switching ala415 to ser in AncLophoSR does not change the ancestral receptor's estrogen-sensitive activity (Fig. S5).

These experiments confirm the importance of the key substitutions l536F, a415W, and h524F in the evolution of constitutive activity. In the genetic background of CgER, the derived states at these sites confer strongly increased constitutive activity and a loss of ligand regulation compared to having the ancestral states, just as they do in AncLophoSR, although there are some subtle differences in their interactions in the two different contexts. Thus, the capacity of these historical substitutions to recapitulate the evolution of constitutive activity is robust to whether or not the genetic background contains the many other sequence changes that occurred along the lineage leading to extant mollusks — an interval of more than 500 million years and over 100 substitutions.

### Structural causes for the effects of key mutations on allosteric regulation

Finally, we sought to understand the structural mechanisms that mediated the functional effects of the four key historical mutations during evolution and their epistatic interactions with each other. Ligand-activated receptors serve as allosterically controlled transcriptional regulators: they exist in a thermodynamic equilibrium, in which the inactive conformation is favored in the absence of ligand and the active conformation is favored in its presence. Examination of the CgER structure suggests that the historical substitutions confer constitutive activity not by filling the ligand cavity per se but by stabilizing the active conformation in the absence of ligand enough to remove allosteric control. The additional restrictive mutations prevented reacquisition of ligand-sensitivity by occluding binding of the ligand and conferring excess stability to the active conformation.

**Figure 6:**
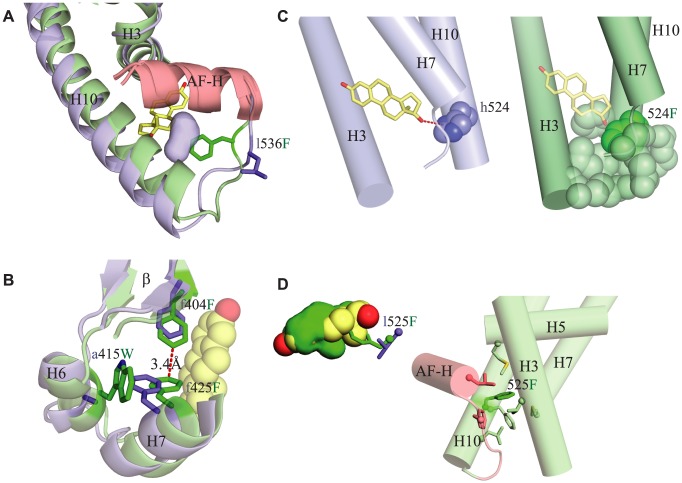
Structural mechanisms by which key mutations contribute to constitutive activity. Each panel shows the CgER crystal structure in green and the AncLophoSR homology modeled structure in blue. Side chains of key residues are shown as sticks; amino acids in AncLophoSR are labeled using lower case letters before the residue number; CgER states are in upper case after the residue number. **A.** Substitution l536F fills a small secondary cavity in the AncLophoSR (blue surface) just below the AF-H (red), improving packing of AF-H with H3 and H10. **B.** Substitution a415W pushes neighboring conserved residue F425 into the ligand cavity, where it packs against F404 and stabilizes the interactions of H6, H7 and the β-sheet. **C.** h524F abolishes a hydrogen bond (red dotted line) to estradiol (yellow) in AncLophoSR. In CgER, the aromatic residue F524 (dark green spheres) packs against several amino acids on H3, H7 and H10 (light green spheres), forming a bridge across the bottom of the pocket. **D.** Substitution l525F occludes the ligand pocket. Left panel: bulky residue F525 occludes the ligand cavity (green) and would clash directly with estradiol (yellow). Right panel: F525 (dark green sticks) also makes van der Waals contacts to numerous residues (light colored sticks), connecting H10 to H3, H5, H7, and AF-H.

Specifically, the large-effect substitution l536F – which potentiates transcriptional activity in the presence or absence of ligand and is required for the evolution of constitutive activity – stabilizes the interaction of the activation function helix (AF-H) with the rest of the protein by improving packing interactions. The small side chain of the ancestral leu 536 leaves a small secondary cavity open within the protein interior – one spatially distinct from the ligand pocket – at the crucial point where AF-H, H3, and H10 meet to stabilize the tertiary structure of the active conformation (Fig. 6A). Replacing leu with the much bulkier Phe fills this cavity and acts as a sort of linchpin that improves packing of these helices against each other, presumably stabilizing the active conformation and providing a structural explanation for this mutation's potentiating effect on activation by the receptor.

Substitution a415W – which has little effect on its own but enhances constitutive activity and abolishes the response to estrogen once F536 is present – stabilizes the active conformation by improving packing interactions and contributes indirectly to occlusion of the ligand cavity. This residue lies outside of the ligand cavity on helix H6, but it interacts with F425, a conserved residue on H7, the side chain of which lines the cavity. Replacing the small side chain of the ancestral a415 with the much bulkier derived Trp causes a clash with F425, which in turn moves directly into the ligand cavity and packs against F404, another conserved residue on the beta-turn. The result is to both occlude the ligand pocket and to result in stronger packing interactions between H6 and H7 and between H7 and the beta-turn, increasing stability along that face of the protein (Fig. 6B). These structural effects are consistent with a415W's effects of preventing activation by hormone and increasing constitutive activity in the absence of ligand.

Substitution h524F – which also does not strongly affect function in isolation but increases constitutive activity when combined with F536 – contributes to the loss of ligand activation and the evolution of constitutive activity more directly. In the estrogen- activated receptors, the ancestral histidine side chain accepts a hydrogen bond from estradiol's 17β-hydroxyl; replacing this residue with the larger, nonpolar Phe eliminates this interaction and causes a clash with the ligand (Fig. 6C), explaining this substitution's negative effect on ligand activation. In addition, Phe's aromatic ring packs strongly against hydrophobic side chains on helices 3 and 6/7, forming a “bridge” across the bottom of the pocket from helix 10 that stabilizes the receptor in the absence of ligand, explaining its enhancement of constitutive activity (Fig. 6C). An aromatic Tyr residue, as found in other mollusk ERs, is anticipated to behave similarly. The redundancy of h524F and a415W presumably occurs because either derived residue is sufficient to clash with ligand, abolishing activation by hormone, and – if F536 is present – to achieve maximal activation in the absence of ligand.

Finally, substitution l525F, when introduced into AncLophoSR, destroys receptor activation whether ligand is present or not. The bulky sidechain of the derived Phe points directly into the ligand cavity, clashing with estrogen and packing against residues on the AF-H loop and helix H3 (Fig. 6D), explaining its deleterious effect on estrogen activation. In CgER, Phe 525 also contributes to constitutive activity, because it serves as a structural hub that makes van der Waals contacts to numerous residues around it, connecting H10 to residues on H3, H7, and AF-H. Why the Phe replacement also eliminates constitutive activity – and does so in all combinations of ancestral and derived states when introduced into AncLophoSR (Fig. 4) – is unclear. The opposite effect of this substitution in AncLophoSR implies that the effects of this residue, which packs against so many structural elements important for activation, depends on the specific position and angle of the backbone and the rotamers at nearby sites.

### Vestigialization of an allosteric switch

The evolution of the mollusk ER can be understood as a vestigialization of the protein's allosteric regulatory mechanism. This process is analogous to the gradual and neutral evolutionary degradation of unused morphological characters – such as the hindlimbs of whales or the eyes of cavefish [44], [45] – which then leave underlying structural traces of their past existence. In this case, the LBD became “stuck” in the active conformation whether or not ligand was bound, due to two large-effect mutations that stabilized the active conformation, shifting the equilibrium towards that conformation even in the absence of ligand. Once allosteric regulation was lost, the architecture that had been required for ligand-dependence – such as the large internal cavity for binding ligand and the dependence of AF-H's position on ligand – degraded further, without apparent consequence for the receptor's transcriptional function or allosteric regulation. Vestiges of this architecture, however, have persisted in the extant mollusk ER in a nonfunctional state since the molluscan ancestor, >500 million years ago [46], as demonstrated by the fact that CgER can regain ligand-dependence by reversing a small number of historical mutations in and around the ligand cavity and AF-H.

One consequence of vestigialization is that regaining the feature becomes more genetically complex than merely reversing its initial loss, because of additional decay in the underlying architecture. In the case of the mollusk ER's allosteric mechanism, two ancient mutations conferred full constitutive activity, shifting the equilibrium towards the active conformation even in the absence of ligand. Once these two mutations were in place, additional mutations further filled the ligand cavity and further stabilized the activation conformation. These mutations caused no apparent functional effect on the receptor's functional output, because the allosteric mechanism was disabled anyway, but they further degraded the underlying architecture of allostery. As a result, restoring the ancestral function into CgER now requires at least two additional “de-vestigializiating” mutations to remove bulky residues from the pocket and partially shift the equilibrium in the absence of ligand back towards the inactive conformation. A similar “ratchet-like” mechanism has been observed during the evolution of other proteins, with substitutions occurring after a functional shift that make reverse evolution to the ancestral structure and function more genetically complex and evolutionarily unlikely than before [16].

If only two mutations were required to trigger the evolution of constitutive activity in the ancestral background, why would the additional redundant/restrictive substitution(s) have evolved and then been conserved? One possibility is that they evolved neutrally, a result consistent with the finding that they have no apparent effect on receptor function. Neither W415 or F525, the potentially redundant mutations, is conserved in all mollusk ERs, a result consistent with at least partial neutrality. In many mollusks, however, these states have persisted over a long time, an observation that could be explained by the fact that reversion to the ancestral states cannot be accomplished with a single nonsynonymous mutation, and the intermediate amino acids may be deleterious. Alternatively, although the derived states have no discernible effects on allosteric regulation or transcriptional activity, they could contribute to other properties, such as folding stability, that might affect function or fitness in certain lineages or environments.

### Protein architecture and evolutionary genetics

Our findings show how the physical architecture of the steroid receptor LBD shaped its evolutionary potential. Allosteric regulation of SRs involves a shift in the thermodynamics of receptor activation upon ligand binding. In the absence of ligand, the inactive conformation is more stable than the transcriptionally active conformation, so the majority of receptor molecules are in the inactive conformation. When the ligand is bound, however, the active conformation is more stable than the ligand-independent conformation, so the presence of ligand drives most receptor molecules into the active form. Structurally, the difference between the two conformations is relatively subtle, primarily involving the ordering and packing of one helix against the protein's body.

Because of the delicate energetic balance among these functionally distinct but structurally similar conformations, relatively small perturbations in the stability of one conformation vis-à-vis the other have the potential to make a receptor active even in the absence of ligand, or inactive even in its presence. Stabilizing the active conformation in the absence of ligand can make it more stable than the inactive conformation and cause the allosteric switch to become stuck in the “on” conformation, resulting in a constantly active transcription factor. Conversely, destabilizing the active conformation relative can make a receptor unable to activate transcription whether or not ligand is present.

Because of the simple physical basis to evolve constitutive activity, the minimal genetic architecture required to trigger such a shift is simple. Just one or a few mutations can shift the thermodynamic equilibrium among states and radically change the protein's capacity to be regulated allosterically. In contemporary SRs, for example, clinical and laboratory single point mutations are known that make nuclear receptors constitutively active by stabilizing the active conformation in the absence of ligand [47]–[52]. Similarly, our findings indicate that during the historical evolution of the mollusk ER, acquiring only two substitutions was sufficient to confer constitutive activation.

The biophysical architecture of allostery therefore influenced the process of genetic evolution in the mollusk ER. Because of the relatively delicate energetics and subtle structural basis of allosteric regulation, a very small number of mutations triggered a large shift in its functional behavior. In this way, the evolving mollusk ER's structural properties influenced the processes of genetic evolution and made the evolution of a radical shift in function relatively simple in genetic terms. Subsequently, the architecture of the receptor's new constitutive activity allowed additional substitutions to make evolutionary reversal to the ancestral function increasingly complex. Taken together, these findings illustrate how the structural basis of protein function shapes genetic evolution, not only by imposing constraints but also by facilitating the emergence of certain radical changes in function.

## Methods

### Phylogenetics and ancestral reconstruction

Alignments of 135 nuclear receptors, identified using BLAST and downloaded from GenBank and the JGI genome browser (Table S2), were made using MUSCLE [53], [54], followed by manual editing. ProtTest [55] was used to determine the best-fit model of evolution (the JTT substitution matrix [56] with gamma-distributed rate variation, a proportion of invariant sites, and observed amino acid frequencies). The phylogeny was inferred using PHYML [57], and statistical support for each node was evaluated by obtaining the approximate likelihood ratio (the likelihood of the best tree with the node divided by the likelihood of the best tree without the node). The ancestral reconstruction was performed using PAML and Lazarus software [58], [59] using the ML tree edited to place the *Strongylocentrotus purpuratus* ERR in the expected position. This tree editing had very little impact on the ancestral state reconstruction. When we compared the ancestors generated from the edited tree to the ancestors generated from the unedited tree, the ancestral lophotrochozoan ER sequence differed at only two residues, neither of which had high levels of support on either tree (in both cases the ML state had PP<0.22, and the second most likely state was identical to the ML state on the other tree) (Table S3). We used the sequence obtained on the edited tree to resurrect the ligand-binding domain (LBD) of the ancestral lophotrochozoan SR (Genbank ID KC261633).

### Structural biology

The CgER LBD (a gift of T. Matusumoto, National Research Institute of Aquaculture, Japan) was subcloned into pMCSG9, which includes a His-tag, MBP, and TEV cleavage site. The CgER/MBP/His protein was expressed in BL21DE3 pLysS cells, and induced with 200 mM IPTG. The protein was purified using a nickel affinity column. The MBP/His fusion was cleaved from the protein using TEV protease, and MBP/His fusion tag was purified from the CgER with a second run on the nickel column. Fractions containing CgER LBD were dialyzed into 150 mM NaCl, 20 mM TrisHCl (pH 7.4) and 2 mM CHAPS, and concentrated to 3 mg/mL. Chemical purity was assessed by SDS-PAGE. Crystals were grown by hanging drop vapor diffusion from solutions containing 1 µl of protein and 1 µl of 25% Peg 4000, 10% glycerol, 01.M TrisHCl (pH 4.8). Crystals were cryoprotected in 25% Peg 4000, 20% glycerol, 01.M TrisHCl (pH 4.8) and were flash-cooled in liquid nitrogen.

Data were collected at 100 K and wavelength of 1 Å the South East Regional Collaborative Access Team at the Advanced Photon Source (Argonne, Illinois, USA), and were processed and scaled with HKL2000 [60]. Molecular replacement [26] using human ERα (1ERE) was used to determine initial phases for CgER. Structures were refined using COOT [61] and Refmac [62]. All residues were either Ramachandran-favored (98.77%) or allowed (1.23%). The structure has been deposited with the Protein Data Bank (PDB 4N1Y).

VOIDOO was used to calculate the probe-occupied volume of the ligand-binding pocket, using a probe radius of 1.4 angstroms [63]. Cavities were calculated 10 times, with molecules rotated to different orientations prior to the VOIDOO cavity calculation. Values are shown as mean ± standard deviation. Pymol (Schrödinger, LLC) was used to construct all structure figures, and LOVOalign was used to calculate the RMSD between human ERα (1GWR) and the CgER LBD (4N1Y) [64]. MODELLER [65], [66] was used to make a homology model of the mutated CgER LBD. The mutated CgER LBD was modelled onto the CgER LBD structure. Ten homology models were created, and the model with the lowest DOPE score was used for structural comparisons.

### Homology modeling of the AncLophoSR structure

A homology model for the AncLophoSR:estradiol complex was generated using the functionally similar human ERα:estradiol complex (1ERE) as a guide. Residue replacements were performed using the program COOT [67] and rotamers that approximated the side chain positions in either human ERα (1ERE). Four regions of the AncLophoSR model were built using CgER (4N1Y) as a guide due to either gaps or insertions in the sequence alignment (Ala19-Thr28, Asp-87-Lys91, Cys105-Met111) or in one case due to a lack of ordered residues in human ERα (Ala146-Asp166). The AncLophoSR aligns without gaps or insertions with CgER, suggesting that these structural differences have no functional impact with respect to estrogen activation. Rotamers were again corrected by hand using COOT. The model was then subject to 500 rounds of energy minimization in the program Phenix software [68] to correct geometry. Structures were rendered for display using Pymol (Schrödinger, LLC).

### Functional characterization

The hinge and LBD of the *Crassostreas gigas* ER were cloned into the pSG5-Gal4DBD vector (a gift of D. Furlow). The resurrected ancestral lophotrochozoan SR LBD sequence was synthesized as a fusion construct containing the hinge domain and C-terminus of the CgER (Genscript, Piscataway, NJ), and cloned into the pSG5-Gal4DBD vector (Fig. S2B). CHO-K1 cells were transfected using Lipofectamine and Plus with 1 ng LBD plasmids, 100 ng of luciferase reporter plasmid (pFRluc) and 0.1 ng of a normalization plasmid (phRLtk). After 4 hours, the transfection mixture was replaced with medium supplemented with stripped serum, and allowed to recover. The cells were treated with 17β-estradiol (Steraloids, Newport, RI) diluted in medium/serum and incubated for 24 hours. Luciferase assays were performed using DualGlo luciferase (Promega, Madison, WI). Mutations were made using QuikChange Lightning Site-directed mutagenesis (Agilent, Englewood, CO), and were verified by sequencing.

## Supporting Information

Figure S1 Nuclear receptor phylogeny indicates that CgER is a mollusk ER. Support is shown as approximate likelihood ratio statistics.
(PDF)

Figure S2 Amino acid sequences and alignment of CgER, HsERα and AncLophoSR. A. The sequences of the CgER and HsERα are aligned to the reconstructed AncLophSR LBD. Mutated amino acid positions are in red and numbered according to the HsERα sequence. Conserved phenylalanine 425 is shown in blue. B. AncLophoSR sequence for ligand binding assays includes the CgER hinge and C-terminus as indicated in green.
(PDF)

Figure S3 Histogram of posterior probabilities of reconstructed sites in AncLophoSR. Many sites in the reconstructed ancestor are highly supported.
(PDF)

Figure S4 Dose responsive activity of AncLophoSR and reversed CgER. A. AncLophoSR is sensitive to estradiol with EC50 = 12 nM. B. CgER is constitutively active and does not respond to hormone (green). When 4 amino acids are reversed (a415W, l524F, l525F, and l536F), the CgER mutant (blue) loses some constitutive activity and gains hormone sensitivity to estradiol, with EC50 = 140 nM.
(PDF)

Figure S5 Alternate ancestral amino acids do not change ancestral function. AncLophoSR (blue) with all 35 alternate states (alt all) is still hormone sensitive, even though it gains ligand independent activation. The functionally important residue 425 has a second-best alternate reconstruction of serine instead of the maximum likelihood state, alanine. When we substituted a415s in the AncLophoSR is was still hormone sensitive. This was also true if we substituted serine into the CgER (green) with ancestral residues at the three other functionally important positions (ahll to shll). Data combined from three experiments with three replicates each; fold activation above vector only control.
(PDF)

Figure S6 Sequence alignment showing substitutions that occurred on the branch between AncLophoSR and AncMollER. There are 79 differences between the two ancestors. Red residues differ between these ancestors and are conserved in all or all but one Mollusk ER, and green residues differ between ancestors, but are not conserved within extant Mollusk ERs.
(PDF)

Figure S7 A derived state present in other mollusk ERs does not change function. Amino acid position 525 is Phe in CgER and Tyr in all other known extant mollusk ERs. A. Substitution of Tyr (magenta) in the CgER (green) structure shows that it also occludes the hormone binding cavity. B. Substitution of Tyr in in all possible combinations in the AncLophoSR LBD does not change the functional results. Green; all four residues in derived state, yellow; three of four are derived, red; two of four derived, purple; one of four derived, blue; all ancestral.
(PDF)

Table S1 Data collection and refinement statistics for the CgER crystal structure.
(PDF)

Table S2 Accession numbers for sequences used in the nuclear receptor phylogeny.
(PDF)

Table S3 Maximum likelihood sequence and site-specific posterior probabilities for the ancestral lophotrochozoan estrogen receptor LBD.
(PDF)
