# Supplementary figures and images for: Vestigialization of an Allosteric Switch: Genetic and Structural Mechanisms for the Evolution of Constitutive Activity in a Steroid Hormone Receptor
Source: PLoS Genet. 2014 Jan 9;10(1):e1004058. doi: 10.1371/journal.pgen.1004058 (PMC3886901; doi:10.1371/journal.pgen.1004058)

Figure S1

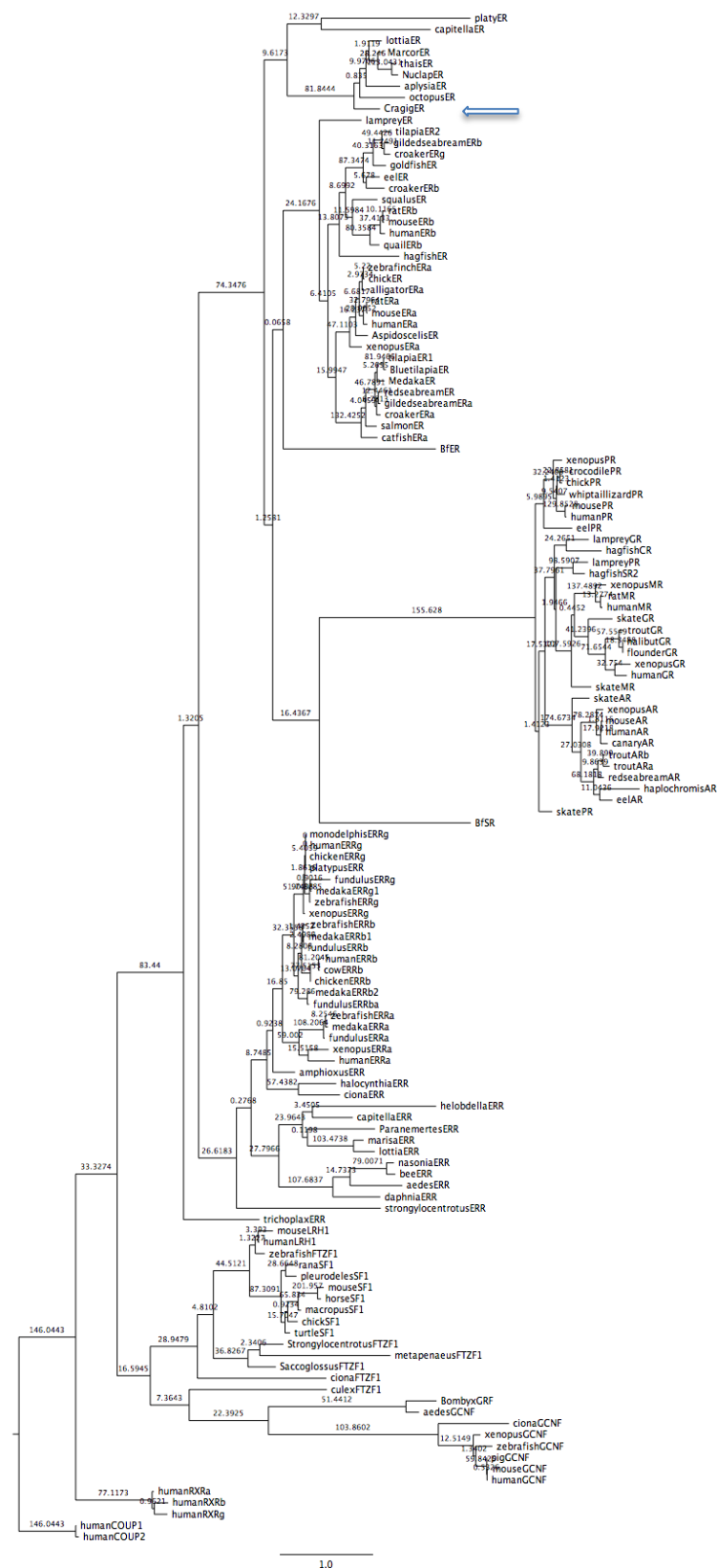

Supplement: Figure S1 — Nuclear receptor phylogeny indicates that CgER is a mollusk ER. Support is shown as approximate likelihood ratio statistics. (PDF) [file pgen.1004058.s001.pdf]

Figure S3

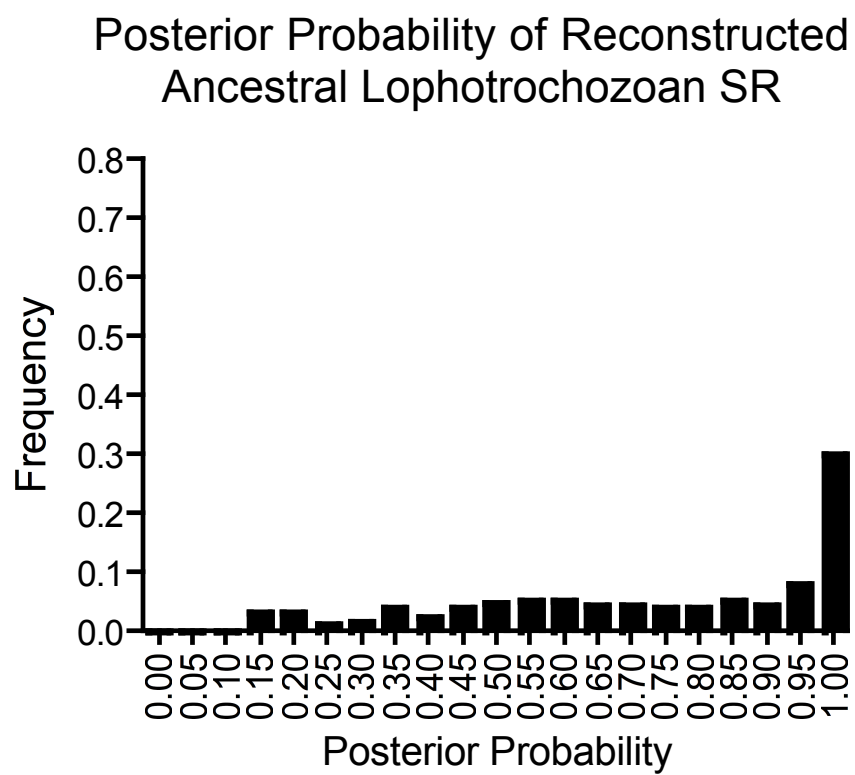

Supplement: Figure S3 — Histogram of posterior probabilities of reconstructed sites in AncLophoSR. Many sites in the reconstructed ancestor are highly supported. (PDF) [file pgen.1004058.s003.pdf]

Figure S4

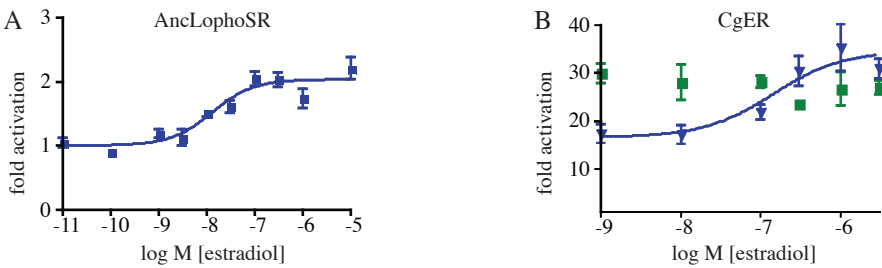

Supplement: Figure S4 — Dose responsive activity of AncLophoSR and reversed CgER. A. AncLophoSR is sensitive to estradiol with EC50 = 12 nM. B. CgER is constitutively active and does not respond to hormone (green). When 4 amino acids are reversed (a415W, l524F, l525F, and l536F), the CgER mutant (blue) loses some constitutive activity and gains hormone sensitivity to estradiol, with EC50 = 140 nM. (PDF) [file pgen.1004058.s004.pdf]

Figure S5

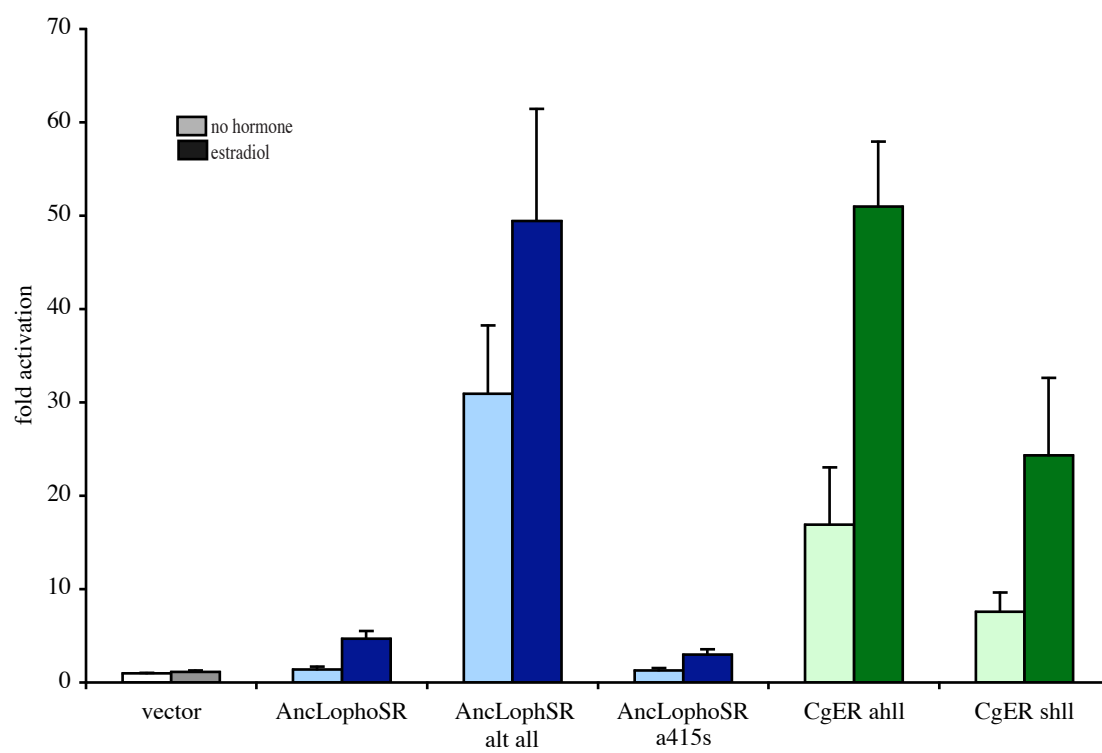

Supplement: Figure S5 — Alternate ancestral amino acids do not change ancestral function. AncLophoSR (blue) with all 35 alternate states (alt all) is still hormone sensitive, even though it gains ligand independent activation. The functionally important residue 425 has a second-best alternate reconstruction of serine instead of the maximum likelihood state, alanine. When we substituted a415s in the AncLophoSR is was still hormone sensitive. This was also true if we substituted serine into the CgER (green) with ancestral residues at the three other functionally important positions (ahll to shll). Data combined from three experiments with three replicates each; fold activation above vector only control. (PDF) [file pgen.1004058.s005.pdf]

Figure S7

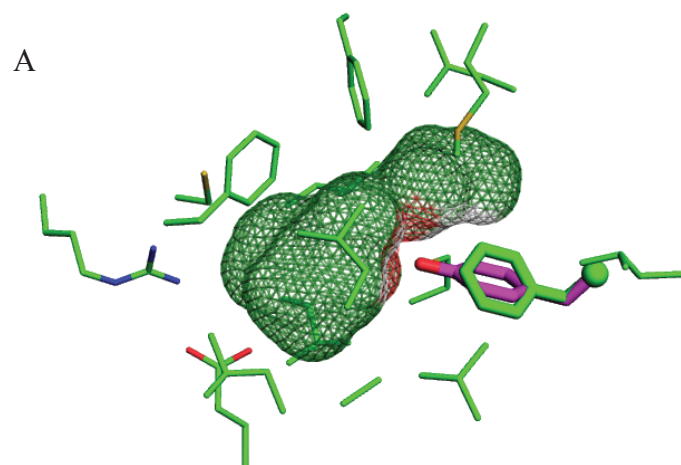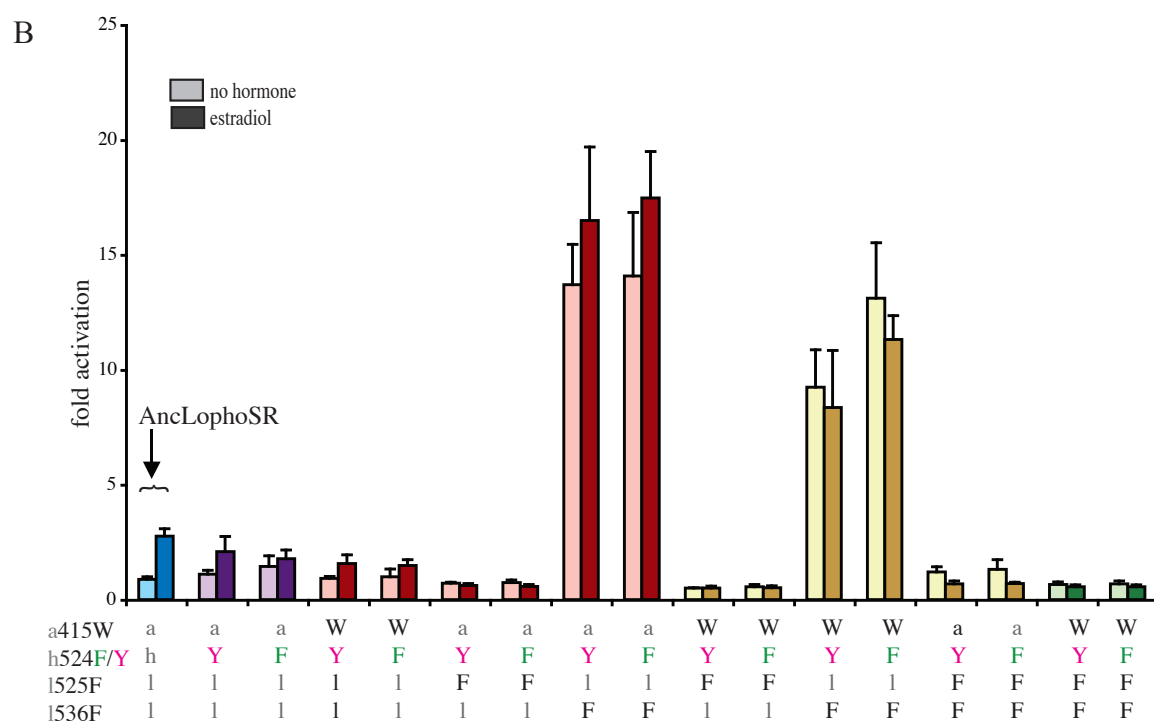

Supplement: Figure S7 — A derived state present in other mollusk ERs does not change function. Amino acid position 525 is Phe in CgER and Tyr in all other known extant mollusk ERs. A. Substitution of Tyr (magenta) in the CgER (green) structure shows that it also occludes the hormone binding cavity. B. Substitution of Tyr in in all possible combinations in the AncLophoSR LBD does not change the functional results. Green; all four residues in derived state, yellow; three of four are derived, red; two of four derived, purple; one of four derived, blue; all ancestral. (PDF) [file pgen.1004058.s007.pdf]
