# Supplementary material for: Vestigialization of an Allosteric Switch: Genetic and Structural Mechanisms for the Evolution of Constitutive Activity in a Steroid Hormone Receptor
Source: PLoS Genet. 2014 Jan 9;10(1):e1004058. doi: 10.1371/journal.pgen.1004058 (PMC3886901; doi:10.1371/journal.pgen.1004058)
Supplement: Figure S2 — Amino acid sequences and alignment of CgER, HsERα and AncLophoSR. A. The sequences of the CgER and HsERα are aligned to the reconstructed AncLophSR LBD. Mutated amino acid positions are in red and numbered according to the HsERα sequence. Conserved phenylalanine 425 is shown in blue. B. AncLophoSR sequence for ligand binding assays includes the CgER hinge and C-terminus as indicated in green. (PDF) [file pgen.1004058.s002.pdf]

Figure S2

A.

```
CgER          248 QTVTILQALNKAALPVLESHHNHGQPPTKVHLLNSLVKLAERELVHLINWAKNVPGYTDL 307
AncLophoSR      1 PANQIISALLKADPPVLYASHDPDLPDTEVHLMTSLIKLADRELVHVINWAKNIPGYTDL  60
HsERα         311 TADQMVSALLDAEPPILYSEYDPTRPFSEASMMGLLTNLADRELVHMINWAKRVPGFVDL 370

                                                               415       425
CgER          308 SLSDQVHLIECCWMELLLLNCAFRSIEHGGKSLAFAPDLVLDRSSWSTVE-MTEIFEQVA 366
AncLophoSR     61 SLNDQVHLLECCWMELLILGLAWRSMDHEGKRLVFAPDLILDREQARVAG-MTEIFDQIL 119
HsERα         371 TLHDQVHLLECAWLEILMIGLVWRSMEHPGK-LLFAPNLLLDRNQGKCVEGMVEIFDMLL 429


CgER          367 AVSEQMMQNHLHKDELLLLQAMVLVNAEVRRLASYNQ--------IFNMQQSLLDAIVDT 418
AncLophoSR    120 AISQQFRQLHLNKEEFVLLKAIVLVNSDVRRLESYSK--------IQELQQNIHDALVDT 171
HsERα         430 ATSSRFRMMNLQGEEFVCLKSIILLNSGVYTFLSSTLKSLEEKDHIHRVLDKITDTLIHL 489

                                                    524/525     536
CgER          419 AQKY---HPDNVRHVPAVLLLLTHIRQAGERGIAFFQRLKSEGVVTFCDLLKEMLDAQ 473
AncLophoER    172 VAKY---HPQNPRRLAQLLLLLPHIRQVSNKGIEHLYSMKSEGAVPLYDLLTEMLDAQ 226
HsERα         490 MAKAGLTLQQQHQRLAQLLLILSHIRHMSNKGMEHLYSMKCKNVVPLYDLLLEMLDAH 547
```

B.

```
AncLophoSR
CYEVGMNKGSQRKERKGSISMSKPAATKRSRADSSDNTVNSTSGSPNPAKSARKSPANQIISALLKADPPV
LYASHDPDLPDTEVHLMTSLIKLADRELVHVINWAKNIPGYTDLSLNDQVHLLECCWMELLILGLAWRSMD
HEGKRLVFAPDLILDREQARVAGMTEIFDQILAISQQFRQLHLNKEEFVLLKAIVLVNSDVRRLESYSKIQ
ELQQNIHDALVDTVAKYHPQNPRRLAQLLLLLPHIRQVSNKGIEHLYSMKSEGAVPLYDLLTEMLDAQDFL
EKKSSNEGD*
```
