# Supplementary material for: Vestigialization of an Allosteric Switch: Genetic and Structural Mechanisms for the Evolution of Constitutive Activity in a Steroid Hormone Receptor
Source: PLoS Genet. 2014 Jan 9;10(1):e1004058. doi: 10.1371/journal.pgen.1004058 (PMC3886901; doi:10.1371/journal.pgen.1004058)
Supplement: Figure S6 — Sequence alignment showing substitutions that occurred on the branch between AncLophoSR and AncMollER. There are 79 differences between the two ancestors. Red residues differ between these ancestors and are conserved in all or all but one Mollusk ER, and green residues differ between ancestors, but are not conserved within extant Mollusk ERs. (PDF) [file pgen.1004058.s006.pdf]

Figure S6

```
AncLophoSR     1 PANQIISALLKADPPVLYASHDPDLPDTEVHLMTSLIKLADRELVHVINWAKNIPGYTDL
AncMollER      1 RSASILEALQKADLPVLESHHNHNIPPTKVHLLNSLIKLADRELVHLINWAKHVPGYTDL

AncLophSR     61 SLNDQVHLLECCWMELLILGLAWRSMDHEGKRLVFAPDLILDREQARVAGMTEIFDQILA
AncMollER     61 SLSDQVHLIECCWMELLLLNCAFRSMEHEGKRLVFAPDLVLDRQQWNVTGMTEIFEQVAA

AncLophSR    121 ISQQFRQLHLNKEEFVLLKAIVLVNSDVRRLESYSKIQELQQNIHDALVDTVAKYHPQNP
AncMollER    121 VSEQMMQYHINKEELLLLQATVLVNAEVRRLASFSKIQDMQQSILDALVDTAQKYHPDNP

AncLophSR    181 RRLAQLLLLLPHIRQVSNKGIEHLYSMKSEGAVPLYDLLTEMLDAQ
AncMollER    181 RHVPSVLLLLTHIRQAGERGIAYFQKLKREGCVTFCDLLTEMLDAQ
```
