# Supplementary material for: Vestigialization of an Allosteric Switch: Genetic and Structural Mechanisms for the Evolution of Constitutive Activity in a Steroid Hormone Receptor
Source: PLoS Genet. 2014 Jan 9;10(1):e1004058. doi: 10.1371/journal.pgen.1004058 (PMC3886901; doi:10.1371/journal.pgen.1004058)
Supplement: Table S1 — Data collection and refinement statistics for the CgER crystal structure. (PDF) [file pgen.1004058.s008.pdf]

Table S1 Data collection and refinement statistics for the CgER crystal structure.

	**CgER LBD**
Resolution (highest shell)	2.60 Å (2.60-2.69 Å)
Space Group	$P2_12_12_1$
Unit Cell Dimensions, Å	a=55.0 b=105.8 c=171.5
	α=β=γ= 90.0
No. of Reflections	26908
R^a_{sym} (highest shell)	14.0% (41.6%)
R (R_{free}) (highest shell)	21.1% (35.0%)
Completeness (highest shell)	90.1% (64.1%)
Ave. Redundance (highest shell)	7.3 (5.2)
I/sigma	15.7 (4.2)
Monomers per assymetric unit	4
No. of protein atoms/AU	7186
No. of waters/ AU	21
$R^b_{working}$ (R^c_{free})	17.0% (24.3 %)
r.m.s. deviations	
Bond lengths, Å	0.009
Bond angles, °	1.302
Average B-factors (Å^2)	
Protein	58.8
Water	51.3

a
Rsym = |I- I|/ |I|, where I is the observed intensity and <I> is the average intensity of several symmetry-related observations.
b
Rworking = ||Fo|-|Fc||/ |Fo, where Fo and Fc are the observed and calculated structure factors, respectively.
c
Rfree = ||Fo|-|Fc||/ |Fo for 5% of the data not used at any stage of the structural refinement.
