# Supplementary material for: Vestigialization of an Allosteric Switch: Genetic and Structural Mechanisms for the Evolution of Constitutive Activity in a Steroid Hormone Receptor
Source: PLoS Genet. 2014 Jan 9;10(1):e1004058. doi: 10.1371/journal.pgen.1004058 (PMC3886901; doi:10.1371/journal.pgen.1004058)
Supplement: Table S2 — Accession numbers for sequences used in the nuclear receptor phylogeny. (PDF) [file pgen.1004058.s009.pdf]

Table S2 Accession numbers for sequences used in the nuclear receptor phylogeny.

sequence name	species name	accession number	NR type
humanCOUP1	Homo sapiens	NP_005645	COUP
humanCOUP2	Homo sapiens	P24468	COUP
alligatorERa	Alligator mississippiensis	AB115909	ER
aplysiaER	Aplysia californica	AY327135	ER
aspidoscelisER	Aspidoscelis uniparens	AB055221	ER
BfER	Branchiostoma floridae	EU371730	ER
BluetilapiaER	Oreochromus aureus	P50240	ER
capitellaER	Capitella capitata	EU497681	ER
catfishERa	Ictalurus punctatus	AAC69548	ER
chickER	Gallus gallus	P06212	ER
CragigER	Crassostreas gigas	BAF45381	ER
croakerERa	Micropogonias undulatus	AAG16713	ER
croakerERb	Micropogonias undulatus	AAG16711	ER
croakerERg	Micropogonias undulatus	AAG16712	ER
eelER	Anguilla japonica	BAA19851	ER
gildedseabreamERa	Sparus aurata	AAD31032	ER
gildedseabreamERB	Sparus aurata	AAD31033	ER
goldfishER	Carassius auratus	AAD26921	ER
hagfishER	Myxine glutinosa	EU439936	ER
humanERa	Homo sapiens	P03372	ER
humanERb	Homo sapiens	BAA24953	ER
lampreyER	Petromyzon marinus	AAK20929	ER
lottiaER	Lottia gigantea	Lotgi1:132166	ER
MarcorER	Marisa cornuarietis	ABI97119	ER
MedakaER	Oryzias latipes	P50241	ER
mouseERa	Mus musculus	P19785	ER
mouseERb	Mus musculus	AAB51132	ER
NuclapER	Nucella lapillus	ABQ96884	ER
octopusER	Octopus vulgaris	Q19AB0	ER
PlatynereisER	Platynereis dumerilii	EU482033	ER
quailERb	Coturnix japonica	AAC36463	ER
ratERa	Rattus norvegicus	CAA43411	ER
ratERb	Rattus norvegicus	AAC52602	ER
redseabreamER	Pagrus major	O42132	ER
salmonER	Salmo salar	P50242	ER
squalusER	Squalus acanthias	AAK57823	ER
thaisER	Thais clavigera	BAC66480	ER
TilapiaER1	Oreochromis niloticus	AAD00245	ER
TilapiaER2	Oreochromis niloticus	AAD00246	ER
xenopusERa	Xenopus laevis	P81559	ER
zebrafinchERa	Taeniopygia guttata	AAB81108	ER
aedesERR	Aedes aegypti	XP_001663736	ERR
amphioxusERR	Branchiostoma floridae	AAU88063	ERR
beeERR	Apis mellifera	XP_392385	ERR
capitellaERR	Capitella capitata	Jgi|Capca1|108381	ERR
chickenERR	Gallus gallus	XP_001235147	ERR
chickenERRb	Gallus gallus	XP_001235147.1	ERR
chickenERRg	Gallus gallus	NP_001007082	ERR
cionaERR	Ciona intestinalis	NP_001071700	ERR

cowERRb	Bos taurus	AAI11278	ERR
daphniaERR	Daphnia pulex	Jgi|dappu1|46682	ERR
dmelanoERR	Drosophila melanogaster	NP_648183	ERR
dpseudoERR	Drosophila pseudoobscura	XP_001354210	ERR
fundulusERRa	Fundulus heteroclitus	ABB80450	ERR
fundulusERRb	Fundulus heteroclitus	ABB80452	ERR
fundulusERRba	Fundulus heteroclitus	ABB80451	ERR
fundulusERRg	Fundulus heteroclitus	ABB80453	ERR
helobdellaERR	Helobdella robusta	jgi|Helro|106750	ERR
humanERRa	Homo sapiens	NM_004451	ERR
humanERRb	Homo sapiens	NP_004443	ERR
humanERRg	Homo sapiens	NP_001438	ERR
lottiaERR	Lottia gigantea	KC261631	ERR
marisaERR	Marisa cornuarietis	ABI97120	ERR
medakaERRa	Oryzias latipes	NM_001104917	ERR
medakaERRb1	Oryzias latipes	NM_001104918	ERR
medakaERRb2	Oryzias latipes	ABQ24187.1	ERR
medakaERRg1	Oryzias latipes	NM_001104919	ERR
monodelphisERRg	Monodelphis domestica	XP_001375146	ERR
nasoniaERR	Nasonia vitripennis	XP_001604033	ERR
paranemertesERR	Paranemertes peregrina	ADM73292	ERR
platypusERR	Ornithorhynchus anatinus	XP_001510517	ERR
strongylocentrotusERR	Strongylocentrotus purpuratus	XP_784483.2	ERR
trichoplaxNR3	Trichoplax adherens	KC261632	NR3
xenopusERRa	Xenopus tropicalis	NP_001072756	ERR
xenopusERRg	Xenopus tropicalis	NM_001100210	ERR
zebrafishERRa	Danio rerio	NM_212955	ERR
zebrafishERRb	Danio rerio	AAS66635	ERR
zebrafishERRg	Danio rerio	NP_998118	ERR
BfSR	Branchiostoma floridae	EU371729	SR
canaryAR	Serinus canaria	AAA17402	SR
chickPR	Gallus gallus	P07812	SR
crocodilePR	Crocodylus siamensi	AAB81722	SR
eelAR	Anguilla japonica	BAA75464	SR
eelPR	Anguilla japonica	BAA89539	SR
flounderGR	Paralichthys olivaceous	O73673	SR
hagfishCR	Myxine glutinosa	ABD46742	SR
hagfishSR2	Myxine glutinosa	ABD46743	SR
halibutGR	Paralichthys olivaceus	BAA25997	SR
haplochromisAR	Astatotilapia burtoni	AAD25074	SR
humanAR	Homo sapiens	NP_000035	SR
humanGR	Homo sapiens	P04150	SR
humanMR	Homo sapiens	P08235	SR
humanPR	Homo sapiens	NP_000917	SR
lampreyGR	Petromyzon marinus	AAK20930	SR
lampreyPR	Petromyzon marinus	AAK20931	SR
mouseAR	Mus musculus	P19091	SR
mousePR	Mus musculus	Q00175	SR
ratMR	Rattus norvegicus	P22199	SR
redseabreamAR	Pagrus major	BAA33451	SR
skateAR	Leucoraja erinacea	Q1KXY2	SR
skateGR	Leucoraja erinacea	Q1KXY4	SR

skateMR	Leucoraja erinacea	Q1KXY3	SR
skatePR	Leucoraja erinacea	Q1KXY1	SR
troutARa	Oncorhynchus mykiss	BAA32784	SR
troutARb	Oncorhynchus mykiss	BAA32785	SR
troutGR	Oncorhynchus mykiss	P49843	SR
whiptaillizardPR	Cnemidophorus uniparens	AAB35740	SR
xenopusAR	Xenopus laevis	AAC97386	SR
xenopusGR	Xenopus laevis	P49844	SR
xenopusMR	Xenopus laevis	Q91573	SR
xenopusPR	Xenopus laevis	AAG42362	SR
culexFTZF1	Culex quinquefasciatus	EDS41949	SF1/FTZF1
chickSF1	Gallus gallus	BAA76713	SF1/FTZF1
cionaFTZF1	Ciona intestinalis	BAE06460	SF1/FTZF1
horseSF1	Equus caballus	NP_001075320	SF1/FTZF1
macropusSF1	Macropus eugenii	Q95L87	SF1/FTZF1
metapenaeusFTZF1	Metapenaeus ensis	AAD41899	SF1/FTZF1
mouseSF1	Mus musculus	P33242	SF1/FTZF1
pleurodelesSF1	Pleurodeles waltl	AAS47030	SF1/FTZF1
ranaSF1	Rana rugosa	BAA36789	SF1/FTZF1
turtleSF1	Trachemys scripta	AAD01975	SF1/FTZF1
zebrafishFTZF1	Danio rerio	AAC60274	SF1/FTZF1
saccoglossusFTZF1	Saccoglossus kowalevskii	ACH68437	SF1/FTZF1
strongylocentrotusFTZF1	Strongylocentrotus purpuratus	XP_791919	SF1/FTZF1
humanLRH1	Homo sapiens	AAD03155	SF1/FTZF1
mouseLRH1	Mus musculus	P45448	SF1/FTZF1
aedesGCNF	Aedes aegypti	XP_001651525	GCNF
bombyxGRF	Bombyx mori	NP_001037015	GCNF
cionaGCNF	Ciona intestinalis	BAE06474	GCNF
humanGCNF	Homo sapiens	AAB50876	GCNF
mouseGCNF	Mus musculus	NP_034394	GCNF
pigGCNF	Sus scrofa	A0P8Z4	GCNF
xenopusGCNF	Xenopus tropicalis	AAH80883	GCNF
zebrafishGCNF	Danio rerio	NP_571331	GCNF
humanRXRa	Homo sapiens	P19793	RXR
humanRXRb	Homo sapiens	P28702	RXR
humanRXRg	Homo sapiens	P48443	RXR
