# Supplementary material for: Vestigialization of an Allosteric Switch: Genetic and Structural Mechanisms for the Evolution of Constitutive Activity in a Steroid Hormone Receptor
Source: PLoS Genet. 2014 Jan 9;10(1):e1004058. doi: 10.1371/journal.pgen.1004058 (PMC3886901; doi:10.1371/journal.pgen.1004058)
Supplement: Table S3 — Maximum likelihood sequence and site-specific posterior probabilities for the ancestral lophotrochozoan estrogen receptor LBD. (PDF) [file pgen.1004058.s010.pdf]

Table S3 Maximum likelihood sequence and site-specific posterior probabilities for the ancestral lophotrochozoan steroid receptor LBD. Functionally characterized residues are highlighted in red.

Human ERα amino acid number	Site#	ML state	PP	Alternate state (if PP>0.25)	PP of alt state	pocket residues
311	1	P	0.159			
312	2	A	0.33	T	0.282	
313	3	N	0.218			
314	4	Q	0.319			
315	5	I	0.582			
316	6	I	0.673			
317	7	S	0.349	A	0.302	
318	8	A	0.746			
319	9	L	1			
320	10	L	0.771			
321	11	K	0.749			
322	12	A	0.919			
323	13	D	0.941			
324	14	P	0.997			
325	15	P	0.99			
326	16	V	0.488			
327	17	L	0.681			
328	18	Y	0.34			
329	19	A	0.521	S	0.397	
330	20	S	0.335			
331	21	H	0.961			
332	22	D	0.804			
333	23	P	0.51	H	0.38	
334	24	D	0.15			
335	25	L	0.153			
336	26	P	0.998			
337	27	D	0.435			
338	28	T	0.976			
339	29	E	0.61			
340	30	V	0.223			
341	31	H	0.393	N	0.38	
342	32	L	0.835			
343	33	M	0.584	L	0.353	
344	34	T	0.513			
345	35	S	0.819			
346	36	L	0.994			*
347	37	I	0.955			*

348	38	K	0.661			
349	39	L	0.999			*
350	40	A	1			*
351	41	D	1			
352	42	R	0.986			
353	43	E	0.999			*
354	44	L	1			
355	45	V	0.989			
356	46	H	0.554			
357	47	V	0.47	I	0.384	
358	48	I	0.999			
359	49	N	0.791			
360	50	W	1			
361	51	A	1			
362	52	K	1			
363	53	N	0.446			
364	54	I	0.842			
365	55	P	1			
366	56	G	1			
367	57	Y	0.668	F	0.332	
368	58	T	0.791			
369	59	D	0.703			
370	60	L	1			
371	61	S	0.993			
372	62	L	1			
373	63	N	0.612			
374	64	D	1			
375	65	Q	0.992			
376	66	V	0.975			
377	67	H	0.879			
378	68	L	1			
379	69	L	0.96			
380	70	E	0.998			
381	71	C	0.572	S	0.399	
382	72	C	0.986			
383	73	W	1			
384	74	M	0.966			*
385	75	E	0.996			
386	76	L	0.734			
387	77	L	1			*
388	78	I	0.596			*
389	79	L	0.66	I	0.25	
390	80	G	0.971			

391	81	L	0.89			*
392	82	A	0.832			
393	83	W	0.972			
394	84	R	1			*
395	85	S	1			
396	86	M	0.532	I	0.289	
397	87	D	0.437	E	0.399	
398	88	H	0.978			
399	89	E	0.311	K	0.252	
400	90	G	0.981			
401	91	K	0.241			
	92	R	0.549	K	0.396	
402	93	L	0.996			
403	94	V	0.508	I	0.312	
404	95	F	1			*
405	96	A	1			
406	97	P	0.999			
407	98	D	0.996			
408	99	L	0.996			
409	100	I	0.521	V	0.335	
410	101	L	0.869			
411	102	D	0.995			
412	103	R	0.89			
413	104	E	0.303			
414	105	Q	0.582			
415	106	A	0.458	S	0.202	
416	107	R	0.565			
417	108	V	0.565			
418	109	A	0.795			
419	110	G	0.956			
421	111	M	0.974			*
422	112	T	0.144			
423	113	E	0.784			
424	114	I	0.997			*
425	115	F	0.644			
426	116	D	0.361	E	0.316	
427	117	Q	0.657			
428	118	I	0.715			*
429	119	L	0.84			
430	120	A	0.489			
431	121	I	0.636	V	0.255	
432	122	S	0.539	A	0.334	
433	123	Q	0.432			

434	124	Q	0.759			
435	125	F	0.99			
436	126	R	0.604			
437	127	Q	0.486	E	0.262	
438	128	L	0.973			
439	129	H	0.379			
440	130	L	0.375	I	0.306	
441	131	N	0.401			
442	132	K	0.644	R	0.339	
443	133	E	0.987			
444	134	E	1			
445	135	F	0.567			
446	136	V	0.876			
447	137	L	0.941			
448	138	L	1			
449	139	K	0.586	R	0.331	
450	140	A	0.972			
451	141	I	0.698			
452	142	V	0.678			
453	143	L	1			
454	144	V	0.721			
455	145	N	1			
456	146	S	0.717	A	0.265	
457	147	D	0.541	E	0.45	
458	148	V	0.915			
459	149	R	0.631			
460	150	R	0.481			
461	151	L	0.994			
462	152	E	0.35			
463	153	S	0.699			
464	154	Y	0.142			
465	155	S	0.175			
466	156	K	0.186			
475	157	I	0.581	V	0.374	
476	158	Q	0.832			
477	159	E	0.259			
478	160	L	0.431	M	0.409	
479	161	Q	0.966			
480	162	Q	0.717			
481	163	N	0.237			
482	164	I	0.744			
483	165	H	0.484	L	0.347	
484	166	D	0.832			

485	167	A	1			
486	168	L	0.999			
487	169	V	0.398			
488	170	D	0.766			
489	171	T	0.768			
490	172	V	0.477			
491	173	A	0.125			
492	174	K	0.415	R	0.285	
493	175	Y	0.154			
497	176	H	0.429	Q	0.276	
498	177	P	0.961			
499	178	Q	0.353	E	0.324	
500	179	N	0.354			
501	180	P	0.81			
502	181	R	0.913			
503	182	R	0.997			
504	183	L	0.625			
505	184	A	0.601			
506	185	Q	0.837			
507	186	L	0.78			
508	187	L	0.999			
509	188	L	0.833			
510	189	L	0.964			
511	190	L	1			
512	191	P	0.813			
513	192	H	0.996			
514	193	I	0.831			
515	194	R	1			
516	195	Q	0.951			
517	196	V	0.558	A	0.297	
518	197	S	0.904			
519	198	N	0.224			
520	199	K	0.536	R	0.442	
521	200	G	0.964			*
522	201	I	0.94			
523	202	E	0.582			
524	203	H	0.979			
525	204	L	0.894			
526	205	Y	0.618			
527	206	S	0.33			
528	207	M	0.531	I	0.337	*
529	208	K	0.984			
530	209	S	0.175			

531	210	E	0.702				
532	211	G	0.773				
533	212	A	0.203				
534	213	V	0.999				
535	214	P	0.996				
536	215	L	0.846				
537	216	Y	0.439		C	0.354	
538	217	D	0.998				
539	218	L	1				
540	219	L	0.996				
541	220	T	0.321				
542	221	E	1				
543	222	M	1				
544	223	L	0.999				
545	224	D	0.83				
546	225	A	0.998				
547	226	Q	0.88				
		Mean PP=	0.7284823				
